# Assessment of the knowledge, attitude and practice of health care workers in Fako Division on post exposure prophylaxis to blood borne viruses: a hospital based cross-sectional study

**DOI:** 10.11604/pamj.2018.31.108.15658

**Published:** 2018-10-12

**Authors:** Che Henry Ngwa, Elvis Akwo Ngoh, Samuel Nambile Cumber

**Affiliations:** 1 Epidemiology and Control of Infectious Diseases, Department of Microbiology and parasitology, University of Buea, Cameroon; 2 Institute of Medicine, Department of Public Health and Community Medicine (EPSO), University of Gothenburg, SE-405 30 Gothenburg, Sweden

**Keywords:** Health care workers, post exposure prophylaxis, blood borne viruses

## Abstract

**Introduction:**

Accidental exposure to blood and body fluid presents a serious public health concern, especially among healthcare workers (HCW) and constitutes a risk of transmission of blood borne viruses. Infections acquired through occupational exposure are largely preventable through strict control measures such as the use of safe devices, proper waste disposal, immunization and prompt management of exposures including the use of Post Exposure Prophylaxis. This study aimed to assess the knowledge, attitude and practice of healthcare workers on post exposure prophylaxis and also determine the factors influencing reporting of occupational exposures among HCW in Fako Division, Cameroon.

**Methods:**

this was a hospital based cross-sectional study conducted from February 2016 to July 2016 involving the administration of questionnaires to 216 health care workers in Fako division. Data collected was analyzed with SPSS version 22 and results presented as percentages and tables. Pearson chi-square test was used to determine statistically significant relationship between different factors with reporting of occupational exposures among health care workers in Fako division.

**Results:**

a high proportion of participants 125(58%) had poor knowledge on Post Exposure Prophylaxis and 131(60.6%) of participants proved to have a positive attitude towards post exposure prophylaxis. 50.9% (110/216) of all participants had at least one occupational exposure with a low uptake 19.1(21/110) of Post Exposure Prophylaxis recorded among participants who were exposed. There was a statistically significant relationship between years of experience (p-value = 0.006, CI= 0.151-0.745) and category of health care worker (p-value= 0.022, CI=0.314-14.215) with reporting of occupational exposure (p-value< 0.05).

**Conclusion:**

this study recorded among participants a poor knowledge on post exposure prophylaxis and poor practice though a majority of study participants had possitive attitude towards PEP. Therefore, a formal training for all health care workers on post exposure prophylaxis to blood borne viruses, strict monitoring and evaluation of health care worker's adherence to standard precautions, adequate reporting of exposures and uptake of post exposure prophylaxis is recommended.

## Introduction

Blood borne viruses (BBV) constitute a variety of infectious agents that can be transmitted through blood and sometimes other body fluids and tissues. Health care workers are at high risk of occupational exposure to blood borne viruses including hepatitis B virus (HBV), hepatitis C virus (HCV) and human immunodeficiency virus (HIV) [1]. Blood and body fluid (BBF) exposures are the most common safety problems among health care workers [2]. The risk of blood borne virus transmission following occupational exposure to blood and body fluids depends on a variety of factors such as the titer of virus in the source patient's blood or body fluid, the type of injury, quantity of blood or body fluid transferred to the health care worker during the exposure and the health care worker's immune status [3, 4]. Occupational exposure to blood and body fluid is a serious public health problem [5] with health care workers at high risk of infection as a result of the high prevalence of infections among patients and increased occupational risk among health care workers due to unsafe practices like careless handling of contaminated needles, unnecessary injections on demand, reuse of inadequately sterilized needles, improper disposal of hazardous waste and overcrowding of hospitals [6, 7]. However, infections acquired through occupational exposures are largely preventable through strict infection control measures such as the use of safe devices, proper waste disposal, immunization and prompt management of exposures including the use of post exposure prophylaxis [8]. Twenty six viruses are known to have caused documented occupational infection following exposure to blood and body fluids (BBF) among health care workers [9, 10] but three viruses alone (HBV, HCV, and HIV) account for most cases of occupational infection in Cameroon because of their prevalence among patients and the severity of the infections they cause. According to the World Health Organization (WHO) Report [11], 2.5% of HIV cases among Health care Workers and 40% of Hepatitis B and C cases among Health care workers worldwide are as a result of occupational exposure. Over 90% of these infections are occurring in low-income countries, and most are preventable [2, 12, 13]. In addition to taking post exposure prophylaxis by health care workers after an exposure, the world health organization recommends the surveillance of occupational accidents, infections due to occupational accidents and uptake of post exposure prophylaxis among exposed health care workers. There exist no data on the prevalence of occupational accidents, the reporting of occupational exposures and the uptake of post exposure prophylaxis among health care workers in Fako division. The absence of such data realized during the first phase of this study(pre-test) further raises concerns about the knowledge, attitude and practices of health care workers in Fako division. According to Mesfin & Kibret [14], Prevention against any disease is proportional to knowledge, attitude and practice of the population. This study therefore aimed to assess the knowledge, attitude and practices of health care workers in Fako Division regarding post exposure prophylaxis to blood borne viruses and also determine the factors influencing the reporting of occupational exposures in health care settings.

## Methods

**Study design:** This was a hospital based cross-sectional study carried out from February 2016 to July 2016 in Fako Division, Southwest region of Cameroon. Fako division is made up of four health districts which includes Limbe health district, Tiko health district, Muyuka health district and Buea health district. This study was conducted in the main public hospital in each health districts and this included Buea regional hospital, Limbe regional hospital, Muyuka district hospital and Tiko district hospital.

**Data collection and analysis:** This study involved the administration of a self-designed questionnaire to three categories of health care workers in Fako division namely physicians, nurses and laboratory technicians/scientist to assess their knowledge, attitude and practice on post exposure prophylaxis to blood borne viruses. These three categories of healthcare workers were chosen based on previous studies demonstrating a high prevalence of occupational accidents among these categories of healthcare workers thereby increasing the possibility of blood borne virus transmission and hence the need to assess their knowledge, attitude and practices regarding post exposure prophylaxis. The questionnaire contained questions on the demographic characteristics of participants and also questions to assess knowledge, attitude and practice of participants. The questionnaire contained both open-ended and closed-ended question to allow for optimum chances of gathering vital data. A pre-test of the questionnaire was done among 35 health care workers consisting of nurses, physicians and laboratory scientistin Fako division who were excluded from the main study. After the pre-test, questions were modified accordingly so as to achieve the desired aim of the study. The pre-tested questionnaires were administered to 216 randomly selected health care workers constituting more than half of the above three categories of health care workers in the hospitals involved in this study. Data collected from the field was entered and analyzed using SPSS version 22. Results were summarized in frequencies, percentages and presented in tables. Assessment of variables for this study was based on WHO recommendations of the process of administering post exposure prophylaxis. The responses to questions aimed at assessing either knowledge, attitude or practice of each health care worker were grouped together and scored. Based on the number of correct responses by each participant, they were categorized as either good (≥ 70%), average (50-69%) or poor (< 50%).The score of attitude of participants was further categorized as positive attitude (> 50%) or negative attitude (< 50%). Statistically significant relationship between years of experience, category of health care worker, knowledge on post exposure prophylaxis with attitude of participants and reporting of occupational exposures to blood and body fluids was investigated using Pearson chi-square test. A p-value < 0.05 was considered statistically significant.

**Ethical consideration:** Ethical approval for the study was obtained from the Faculty of Health Science Ethical Review Board University of Buea, Cameroon. Administrative clearance was obtained from the regional delegation of public health. Written permission was obtained from the directors of the hospitals where the study was carried out. Finally, written consent was obtained from all participants and confidentiality of participants was ensured by using anonymous questionnaires in addition to the fact that the study database was only accessible to the principal investigator.

## Results

A total of 148 (68.5%) females and 68 (31.5%) males responded in the study. Most of the participants 160 (74.1%) were of age between 26 years and 45 years. More than half of the study participants 126 (58.3%) comprised of nurses and more than half of the study participants 120 (55.6%) had been working for more than 5 years (Table 1).

**Table 1:** Demographic characteristics of participants

Demographic characteristics		
Age of respondents	**Category**	**Number (%)**
	< 25years	24 (11.1)
	26years- 45years	160 (74.1)
	>46years	32 (14.8)
Nationality	Cameroonian	208 (96.3)
	Nigerian	8 (3.7)
Gender	Male	68 (31.5)
	Female	148 (68.5)
Category of HCW	Lab Scientist	68 (31.5)
	Nurse	126 (58.3)
	Physician	22 (10.2)
Years of experience	less than 3years	44 (20.4)
	between 3-5yearrs	52 (24.1)
	greater than 5years	120 (55.6)

**Knowledge:** Majority of participants across all the category of health care workers (72.7% of physicians, 52.3% of nurses and 64.7% of laboratory technicians/scientist) had a highknowledge on the transmission of blood borne viruses in health care settings. Regarding knowledge on PEP, a higher proportion of all study participants 125 (58%) had poor knowledge on PEP while 27 % (58/216) of study participants had good knowledge on post exposure prophylaxis. Majority of nurses 81 (64.3%) and laboratory scientist 42 (61.7%) had poor knowledge on PEP (Table 2).

**Table 2:** Knowledge on post exposure prophylaxis per category of HCW

	Category of HCW (%)	
	Lab Technicians (%)	Nurses (%)	Physicians (%)
Low knowledge	42 (61.7)	81 (64.3)	2 (9.1)
Average knowledge	11 (16.2)	21 (16.7)	0 (0.0)
High knowledge	15 (22.1)	24 (19.0)	20 (90.9)

**Attitude:** More than half 131 (60.6%) of study participants proved to have a positive attitude towards post exposure prophylaxis while 85 (39.4%) had a negative attitude towards post exposure prophylaxis with majority of nurses showing a more positive attitude (62.7%) as compared to lab technicians and physicians (Table 3). There was no statistical significant relationship between years of experience of health care worker (p-value = 0.681, CI = 0.68 - 0.699), age (p-value = 0.951, CI = 0.971-0.977 ), category of health care worker (p-value = 0.719, CI = 0.707-0.725 ), number of exposures experienced by the healthcare worker (p-value = 0.819, CI = 0.835-0.850) and knowledge on post exposure prophylaxis (p-value = 0.444, CI = 0.446-0.465) with attitude of health care workers towards post exposure prophylaxis.

**Table 3:** Attitude of participants towards post exposure prophylaxis

		Attitude	
		Negative attitude (%)	Positive attitude (%)
Category of HCW	Lab Technician	28 (41.2)	40 (58.8)
	Nurses	47 (37.3)	79 (62.7)
	Physician	10 (45.5)	12 (54.5)
	Male	30 (44.1)	38 (55.9)
Gender	Female	55 (37.2)	93 (62.8)

**Practice of study participants:** 50.9% (110/216) of study participants reported to have had at least one occupational exposure from January 2015. Among the 110 participants who had at least an occupational exposure to blood and body fluid, 47% (52/110) reported that they have had one exposure, 44% (48/110) had between 2 to 5 exposures and 9% (10/110) had more than 5 exposures from January 2015 to April 2016. The highest proportion of exposures within the study period was recorded among physicians 63.6% (14/22), followed by nurses 57.1% (72/126) and lastly lab technicians 35.3 % (24/68). Among the 110 participants who experienced at least an occupational exposure, 68 (61.8%) verbally reported the exposures and none of the exposures were documented as recommended by WHO. The 38.2 % of exposed participants who did not report their exposures gave reasons which were same as responses provided by all participants to the question of what might cause you not to report an exposure. These responses are summarized in the table that follows (Table 4). Among the 68 participants who actually reported an occupational exposure, 21 (30.8%) received post exposure prophylaxis. There was a statistically significant (p-value < 0.05) relationship between years of experience, category of health care worker and frequency of exposure with reporting of occupational exposure using Pearson chi-square test (Table 5).

**Table 4:** Reasons why participants might not report an exposure

	Category of Health care worker		
	Lab Technicians (%)	Nurses (%)	Physicians (%)
Fear of being stigmatized and absence of routes of reporting	24(11.1)	32(14.8)	4(1.9)
Fear of being stigmatized	10(4.6)	40(1.5)	4(1.9)
Absence of routes of reporting	32(14.8)	40(18.5)	14(6.5)
I did not know I was to report	2(0.9)	10(4.6)	0(0.0)
It was not necessary to report	0(0.0)	2(0.9)	0(0.0)
Others	0(0.0)	2(0.9)	0(0.00)

**Table 5:** Factors related with reporting of occupational exposures

variables	Pearson chi square value	p-value	confidence interval
Gender of participants	0.629	0.428	0.331-1.599
Frequency of exposure	5.296	0.021	1.138 - 5.641
Category of Health care worker	7.647	0.022	0.314 - 14.215
Knowledge on PEP	0.455	0.500	0.351-1.667
Experience of Health care worker	7.415	0.006	0.151-0.745
Age	0.693	0.405	0.226-1.828

## Discussion

More than half of study participants 125 (58%) had poor knowledge on PEP and though a greater proportion of health care workers had good knowledge on the transmission of blood borne viruses in health care settings, knowledge on PEP was poor among health care workers in Fako division. This result is similar to results recorded by another study conducted in Lagos, Nigeria where only 15.3% of participants knew the correct duration of PEP for HIV [13].The poor knowledge on post exposure prophylaxis recorded among participants could be due to lack of interest in the topic of post exposure prophylaxis on the part of healthcare workers. Furthermore, the poor knowledge on PEP is partly due the fact that the subject of PEP is not incorporated to a considerable extent in the curriculum and training of healthcare workers. This result is an indication to the fact that much is needed to be done to improve the knowledge of health care workers on post exposure prophylaxis to blood borne viruses. Physicians had good knowledge on PEP as opposed to laboratory workers and nurses. This is probably because physicians undergo a longer period of training than Nurses and laboratory scientist/technicians coupled with numerous in-service seminars and workshops organized for physicians. This study revealed a positive attitude among 131 (60.6%) of study participants. This is similar to a study in Ethiopia where more than half (55.6%) of the respondents had a positive attitude [15] but contrary to another study where less than half of the study participants had a positive attitude [16]. The result show that 50.9% (110/216) of all respondents had at least one occupational exposure within the study period. This high number of exposures can be explained by the fact that HCW fail to adhere to universal precautions and also due to unsafe practices in health care settings. This is similar to results obtained in a study among medical students in Yaoundé [17]. This similarity could be an indication to the fact that infection control measures are not treated with the seriousness it deserves during the training of health care workers. Among the 110 who experienced an exposure, 68 (61.8 %) said they reported the exposures this is contrary to a study carried out among medical students where only 42% of the exposures were reported [17]. Although a fairly good number of participants said they reported their exposures, this study revealed that the reporting of occupational exposure among health care workers in Fako division remain inadequate since none of the exposures were documented as recommended by the world health organization. This could be due to absence of a route of reporting or absence of a standardized form for reporting of occupational accidents blood and body fluid among health care workers in Fako division. This reason is largely supported by the high proportion of health care workers indicating the lack of a route of reporting as the reason why they did not report their exposure. The lack of data on exposures to blood and body fluids among healthcare workers in Fako division further serves as a confirmation. World health organization recommends the uptake of PEP after an occupational exposure to blood or body fluid. In this study, approximately 30.8% (21/68) of the participants who reported an exposure actually took PEP. This constitutes only about 19% of the total participants who experienced an exposure within the study period. This low uptake of PEP is consistent with other studies in Ethiopia where only about 13% of study participants took PEP [14]. This is in contrast to another study carried out in Ethiopia where more than 60% of exposed participants took PEP [7]. The low uptake of PEP in this study is partlydue to side effects associated with the drugs administered as PEP especially those administered as PEP for HIV. This result is consistent with results of a previous study which also recorded a low uptake with side effects of the drugs administered reported as one of the major reasons among participants [18]. Low uptake of PEP can also be explained by the low knowledge of participants on PEP recorded among health care workers in Fako division. The findings of this study is of clear relevance and though this study is subjected to recall bias in that it might have been difficult for participants to remember their exposure status, findings of similar studies like this study have been shown to be valid and clearly adds to existing literature.

## Conclusion

Overall, this study recorded poor knowledge on post exposure prophylaxis among health care workers in Fako division and though majority of study participants had good knowledge on the transmission of blood borne viruses in health care setting and a favorable attitude towards post exposure prophylaxis, knowledge of participants on post exposure prophylaxis was poor. A high rate of occupational exposures, inadequate reporting of occupational exposures and Low uptake of post exposure prophylaxis was also recorded among health care workers in Fako division. Therefore, training of health care workers on infection control measures at work and the creation of a standard form for adequate reporting of occupational exposures coupled with strict monitoring and evaluation of health care worker's adherence to standard precautions, adequate reporting of exposures and uptake of post exposure prophylaxis is recommended.

**Weaknesses:** This study fails to look at the gaps in knowledge and practice on post exposure prophylaxis to a specific disease, coupled to the fact that the results of this study cannot be generalized to all health care workers.

**Recommendation:** We recommend an in-depth qualitative study aimed at better understanding the challenges and barriers of health care workers towards the uptake of prophylaxis after an exposure to blood and body fluids. We also recommend such studies to be conducted at regional and national level so as to clearly identify the gap in knowledge and challenges towards uptake of post exposure prophylaxis among health care workers at different levels. This would ease planning and direct interventions aimed at addressing this problem at each level.

### What is known about this topic

- Health care workers are at risk of blood borne virus infections due to occupational exposure to blood and body fluids;
- Post exposure prophylaxis is an effective means of preventing the development of an infection after an exposure to infectious blood or body fluid.

### What this study adds

- This study reveals the absence of a route of reporting of occupational exposures to blood and body fluids in Fako division and further expresses the need for documenting such exposures among health care workers;
- This study also brings into light some reasons why healthcare workers do not report occupational exposures to blood and body fluids and also further identifies some drawbacks to the uptake of post exposure prophylaxis after an exposure to blood and body fluid among health care workers in Fako division.

## Competing interests

The authors declare no competing interest.
